# A thorough investigation into the correlation between migraines and the gut microbiome: an in-depth analysis using Mendelian randomization studies

**DOI:** 10.3389/fneur.2024.1356974

**Published:** 2024-07-02

**Authors:** Xuege Zang, Yongkun Du, Mengshu Jiang, Shiyao Zhou, Libo Wang, Xuemei Han

**Affiliations:** ^1^Department of Critical Care Medicine, China-Japan Union Hospital of Jilin University, Changchuan, Jilin, China; ^2^Unity Health Toronto, Toronto, ON, Canada; ^3^Department of Neurology, China-Japan Union Hospital of Jilin University, Changchuan, Jilin, China

**Keywords:** Mendelian randomization, meta-analysis, migraine, gut microbiome, migraine with aura, migraine without aura

## Abstract

**Objective:**

A growing body of evidence underscores a significant association between neurological disorders, particularly migraines, and the gut microbiota. However, a research gap persists in understanding the cause-and-effect dynamics between these elements. Therefore, we employed robust methodologies aimed at thoroughly exploring the causal relationship between the gut microbiome and migraines.

**Methods:**

Employing bidirectional Two Sample Mendelian Randomization (TSMR) analysis, we investigated the causal association between the composition of the gut microbiota and migraines. Data summarizing the relationship between gut microbiota and migraines were extracted from one or more genome-wide association studies. The TSMR analysis employed five methods to assess the correlation between the gut microbiota and migraines, with the inverse variance-weighted method serving as the primary approach for analyzing causal links. Sensitivity analyses were applied to address horizontal pleiotropy and heterogeneity. Simultaneously, a meta-analysis was performed to strengthen the robustness of the findings. Additionally, a reverse TSMR was carried out to explore potential occurrences of reverse causal relationships.

**Results:**

The ongoing TSMR analysis identified a collection of 14 bacterial taxa connected to migraines. Among these, 8 taxa exhibited a protective effect, while 5 taxa had a detrimental impact, and 1 taxon maintained a neutral relationship. The reverse Mendelian randomization analysis highlighted stable outcomes for only one bacterial taxonomic group.

**Conclusion:**

The study confirms a causal relationship between the gut microbiota and migraines, offering a new perspective for migraine research. Strategically targeting specific bacterial taxa with dysregulation may be effective in both preventing and treating migraines, thus opening new avenues for therapeutic strategies.

## 1 Introduction

Migraine, widely recognized as a prevalent neurological challenge with a significant impact on human well-being (1, 2), is categorized by the International Headache Society into two principal types: those accompanied by aura and those without (3). While the intricate pathological details remain elusive, there exists a consensus among researchers concerning the significant involvement of the trigeminal nervous system in the pathophysiology of migraines (4–7).

Numerous studies have explored an association between migraines and disorders of the digestive system, such as irritable bowel syndrome and gastroesophageal reflux (8–10). The increasing recognition of how dietary choices impact migraine episodes sparks consideration of a potential link to the microbiota in the gastrointestinal tract (11, 12). As our understanding deepens on the role of microbiota in neurological conditions like Alzheimer’s and Parkinson’s diseases (13, 14), attention is directed toward its potential interplay with migraines, involving the gut-brain axis (15).

The gut-brain axis, a bidirectional link between the gut and the brain, involves the gastrointestinal tract exerting influence on the central nervous system through various mechanisms (15, 16). Components contributing to this intricate relationship include the immune system, hormonal activity, inflammatory mediators, neuropeptides, and the dynamic world of gut microbiota (16–18). The microbial community not only plays a pivotal role in providing essential nutrients and safeguarding gut integrity but also affects pain perception through intricate endocrine and metabolic signaling pathways (19, 20). Recent research suggests a potential causal association between the gut microbiome and the onset of migraines, highlighting specific bacterial taxa such as the genus Coprococcus3 and genus Anaerotruncus (21). Nevertheless, it is imperative to emphasize the limited scope of these studies, necessitating future research endeavors to authenticate and illuminate the underlying mechanisms.

The ongoing research within this conceptual framework aims to unravel the intricate association between gut microbiota and migraines. Using Mendelian randomization (MR) as a methodological tool and genetic variations as instrumental variables, this study aimed to investigate the potential cause-and-effect relationship between variables such as lifestyle choices or the composition of gut microbiota and the occurrence of specific diseases (22, 23). By leveraging the inherent randomness in the inheritance of genotypes from parents to offspring, MR aims to mitigate the impact of confounding variables that might distort results in observational studies. This enhances the reliability of establishing causal relationships without being influenced by biases from reverse causation (24).

In the pursuit of a comprehensive evaluation of the evidence surrounding the causal relationship between gut microbiota and migraines, we conducted a systematic review of existing Mendelian Randomization (MR) studies Due to the limited literature in this specific domain, we performed a re-examination using consolidated summary statistics from the FinnGen study and other publicly available Genome-Wide Association Studies (GWAS). Subsequently, we conducted a meta-analysis to compile and present a concise overview of the findings derived from the MR analyses.

## 2 Materials and methods

Investigating the potential association between gut microbiota and migraines necessitated a comprehensive examination using TSMR analysis. To enhance the credibility of the findings, we performed a subsequent meta-analysis. Information for this investigation was sourced from diverse sources including published articles and digital repositories, with strict adherence to the MRSTROBE checklist ensuring a thorough investigation (25).

### 2.1 Exposure data

The extraction of instrumental variables related to human gut microbiota involved navigating the MiBioGen Alliance website,^1^ established by Kurilshikov et al. in 2021. This consortium predominantly comprises extensive data, including whole-genome genotype and 16S fecal microbiota from 24 cohorts, involving a total of 18,340 individuals (26). In our investigation, we conducted a meta-analysis, specifically focusing on autosomal human genetic variation and its association with the gut microbiome. The study also investigated the changes in microbiome composition and methodological variations in microbiome data. Employing a standardized pipeline, our objective was to elucidate the specific loci responsible for microbial traits (mbTL). These include genetic loci influencing the relative abundance of microbial taxa (mbQTL) or their presence (microbial binary trait loci, or mbBTLs). This comprehensive analysis included 211 taxa, extending across 35 families, 20 orders, 16 classes, 9 phyla, and 131 genera.

### 2.2 Outcome data

We obtained summary data on migraines from three distinct studies. The primary dataset, centered on migraines, originated from the FinnGen study, which commenced in Finland in 2017, aiming to explore novel targets and methodologies for diverse diseases through genetic research. We retrieved Version 9 data, released on 11 May 2023, from their official website.^2^ This dataset comprised 18,477 migraine cases and 287,837 controls, with an average onset age of 40.27 years, predominantly among females (82.07% of cases). Migraine cases were identified using International Classification of Diseases (ICD) codes from the 8th, 9th, and 10th editions. Specifically, summary data for migraines with aura encompassed 7,917 cases and 287,837 controls, while migraines without aura involved 6,730 cases and 287,837 controls.

The second dataset originated from a study conducted by Choquet et al. (27). In this investigation, data were obtained from the Genetic Epidemiology Research on Adult Health and Aging (GERA) cohort (71,602 individuals, including 11,320 migraine cases) and the UK Biobank (UKB) cohort (482,967 individuals, with 17,532 migraine cases). In both cohorts, the predominant ancestry was European, constituting 94.19 and 81.53%, respectively. Notably, in the UK Biobank, there is a significant 75.30% overlap between the exposure dataset and our study sample, minimizing bias to approximately 1% (28).

The third study is grounded in the research of Dönertaş et al. (29). This study identified four distinct disease clusters among 116 diseases in the UK Biobank data. The clusters were delineated based on the age of onset, confirming genetic associations with age-related diseases through data analysis. Access to data for this study can be obtained in the GWAS Catalog (GCST90038646), including 13,971 migraine cases and 470,627 controls. A comprehensive presentation of information from the outcome databases is provided in Table 1.

**Table 1:** Characteristics of outcome GWAS datasets.

Trait	Study	Data source	Sample size
Any migraine	FinnGen (Release 9)	FinnGen study	18,477cases and 287,837 controls
MA	FinnGen (Release 9)	FinnGen study	7,917cases and 287,837controls
MO	FinnGen (Release 9)	FinnGen study	6,730 cases and 287,837controls
Any migraine	Choquet et al. (27)	UK biobank	17,532 cases and 465,435 controls
		GERA cohort	11,320 cases and 60,282 controls
Any migraine	Donertas et al. (29)	UK biobank	13,971 cases and470,627 controls

GERA, Genetic Epidemiology Research in Adult Health and Aging; GWAS, genome-wide association study; MA, migraine with aura; MO, migraine without aura.

### 2.3 Instrumental variable selection

**Figure 1:**
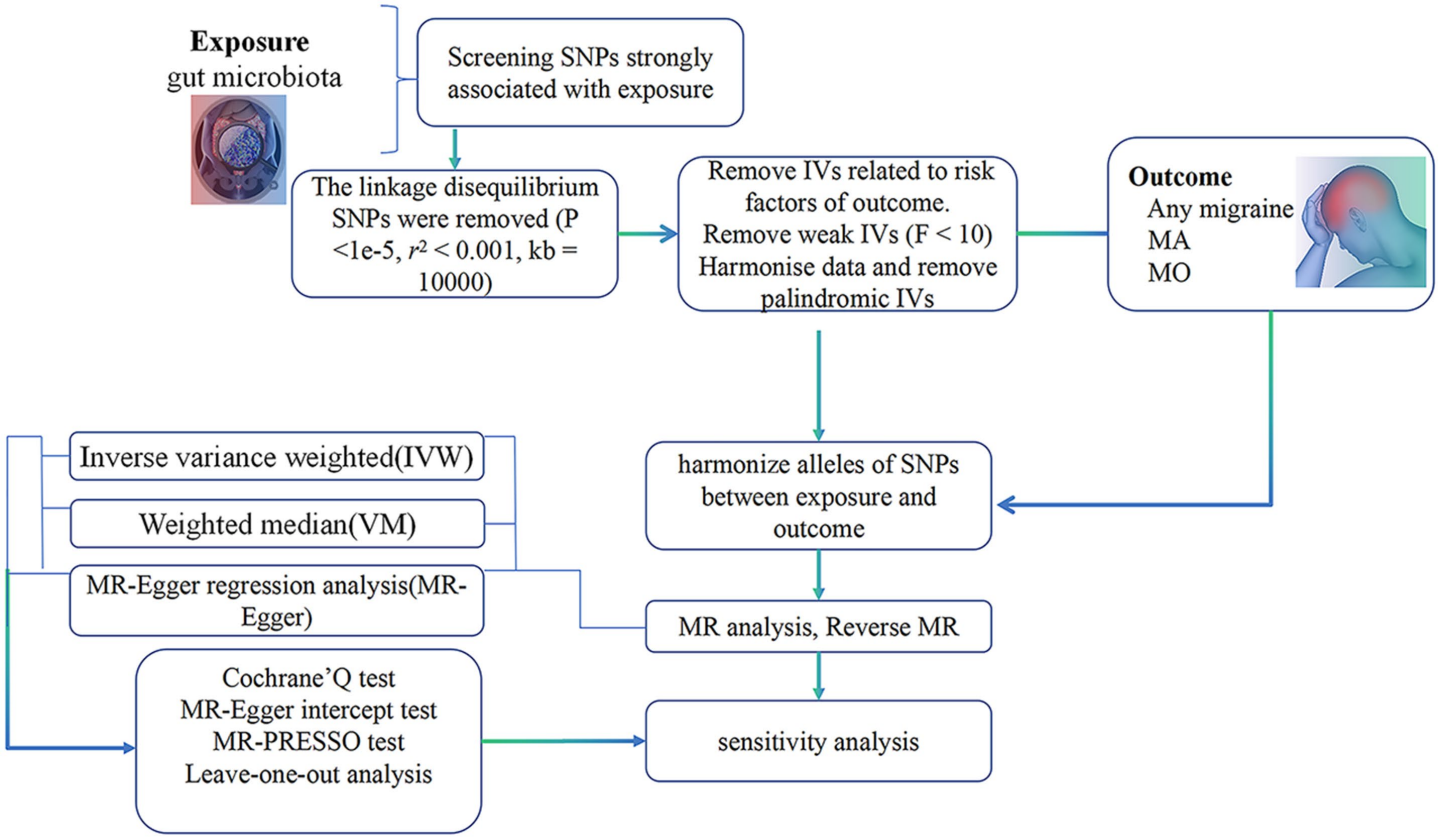
Summary of current Mendelian randomization processes. MA, migraine with aura; MO, migraine without aura.

**Figure 2:**
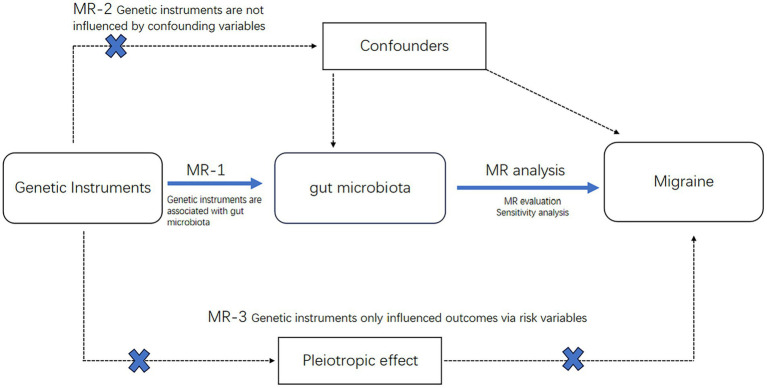
Mendelian randomization of three hypotheses.

The procedural sequence of the investigation is illustrated in Figure 1. To explore the potential causal relationship between gut microbiota and migraines, we conducted a Two-Sample Mendelian Randomization (TSMR) analysis During the selection of instrumental variables, we adhered to three essential three essential prerequisites to ensure the reliability of the outcomes (30) (Figure 2):

1. A fundamental requirement is a close association between gut microbiota and the instrumental variables (IVs) ultimately incorporated.
2. There must be no reciprocal dependence between the included IVs and confounding factors that may affect both the categorization of gut microbiota and migraines.
3. Migraines should be solely influenced by the categorization of gut microbiota through the IVs, without any indication of horizontal pleiotropy.

To explore the causal impact of human gut microbiota on migraines and avoid overlooking potentially significant findings, we set a correlation threshold of 1 × 10^−5^. This choice was deliberate, especially in cases where SNP selection fell below the standard threshold of 5 × 10^−8^ (26). Furthermore, in the context of various MR investigations related to the gut microbiota, the threshold of 1 × 10^−5^ is considered optimal, aimed to increase the number of SNPs meeting the criteria for subsequent analysis (31, 32).

To ensure no linkage disequilibrium among instrumental variables (IVs) associated with gut microbiota, a clumping procedure was implemented (r^2^ < 0.001, clumping window size = 10,000 kb), leveraging European 1,000 Genomes Project sequencing data as the reference panel. Palindrome SNPs were removed during the allele harmonization process to eliminate any distortion in chain orientation or allele coding.

Following this, the F-statistic for each SNP was computed to assess its association strength with gut microbiota. The F-statistic formula is F = R^2^*(n − k − 1)/(k*(1 − R^2^)), where “R^2^” signifies explained variance, “n” denotes the exposure sample size, and “k” represents the number of SNPs (33). SNPs with an F-statistic below 10 were considered weak instrumental variables and subsequently excluded.

Afterward, MR-PRESSO tests and MR-Egger regression tests were employed to address horizontal pleiotropy and detect outliers. MR-PRESSO, recognized for its efficacy in detecting horizontal pleiotropy (34), involved calculating the *p*-value for overall horizontal pleiotropy for each SNP. SNPs were then arranged based on ascending *p*-values from the MR-PRESSO outlier test, and one SNP at a time was systematically removed. After each removal, the MR-PRESSO global test was conducted on the remaining SNPs, until the *p*-value of the global test was no longer significant (*p* > 0.05). Only SNPs that passed through this meticulous filtering process were considered for subsequent MR analysis. All these procedures were performed using the “Two Sample MR” R package (version 0.5.7).

### 2.4 Statistical analyses

To investigate the correlation between gut microbiota and migraines, we employed five techniques within the “Two Sample MR” toolbox. These included Inverse Variance Weighted (IVW) examination (35), MR-Egger regression (36), Weighted Median Estimate (WM) (37), Simple mode, and Weighted mode. Existing scholarly literature emphasizes the superiority of IVW as the primary analytical instrument (37). IVW produces reliable outcomes under the assumption of no horizontal pleiotropy, a condition duly validated (35). The MR-Egger method identifies potential breaches in the instrumental variable selection assumption (38), considering a MR-Egger intercept *p*-value > 0.05 as acceptable. SNP heterogeneity was assessed using Cochran’s Q test, with a *p*-value < 0.05 signifying significant diversity. The “RadialMR” R package (version 1.0) was employed to identify anomalous SNPs and rectify results in cases of significant heterogeneity (39). Horizontal pleiotropy was examined using MR-Egger intercept and MR-PRESSO, with *p* > 0.05 signifying its nonexistence (34, 40). The leave-one-out method identified individual SNP impact on the IVW total effect and established the directionality of the causal relationship (41).

A *p*-value < 0.05 was considered statistically significant. To enhance result robustness, Bonferroni correction was implemented at each taxonomic level (phylum, class, order, family, genus). The Bonferroni-corrected significance threshold was computed as 0.05 divided by the effective number of independent bacterial taxa at each corresponding taxonomic level: genera (0.05/131, 3.81 × 10^−4^), families (0.05/35, 1.4 × 10^−3^), orders (0.05/20, 2.5 × 10^−3^), classes (0.05/16, 3.1 × 10^−3^), and phyla (0.05/9, 5.5 × 10^−3^) (42). *p*-values ranging from 0.05 to the corrected threshold denoted nominal causal effects.

In conclusion, a meta-analysis of IVW results for comparable migraine outcomes was executed using the “meta” R package (version 6.5-0). In instances of heterogeneity (*p* < 0.05), a random-effects model was engaged; otherwise, a fixed-effects model was applied. All statistical analyses were executed in R (version 4.3.1).

### 2.5 Reverse MR analyses

Considering the potential impact of host diseases on gut microbiota, we conducted a reverse MR analysis. Initially, each of the five migraine databases served as individual exposures, with the gut microbiota designated as the outcome. The ensuing MR analysis yielded significant findings. Differing from the earlier phase, we adjusted the threshold for tool variable selection to *p* < 5 × 10^−8^. All other parameters were maintained in accordance with the prior iteration, and the outcomes underwent both Bonferroni correction and sensitivity analysis for validation.

## 3 Results

### 3.1 TSMR analysis

#### 3.1.1 SNP selection

Following pre-established criteria, the selection of instrumental variables (IVs) resulted in the categorization of SNPs for comprehensive analysis within the Two-Sample Mendelian Randomization (TSMR) framework, shedding light on the intricate relationship between gut microbiota and migraines. Detailed information can be found in Supplementary Tables S1–S5. All SNPs considered in the analysis exhibited F-statistics surpassing 10, confirming the exclusion of weak IVs (Supplementary Tables S1–S5). After harmonization, the count of SNPs associated with each pair of bacterial taxa and migraines exceeded 3.

**Figure 3:**
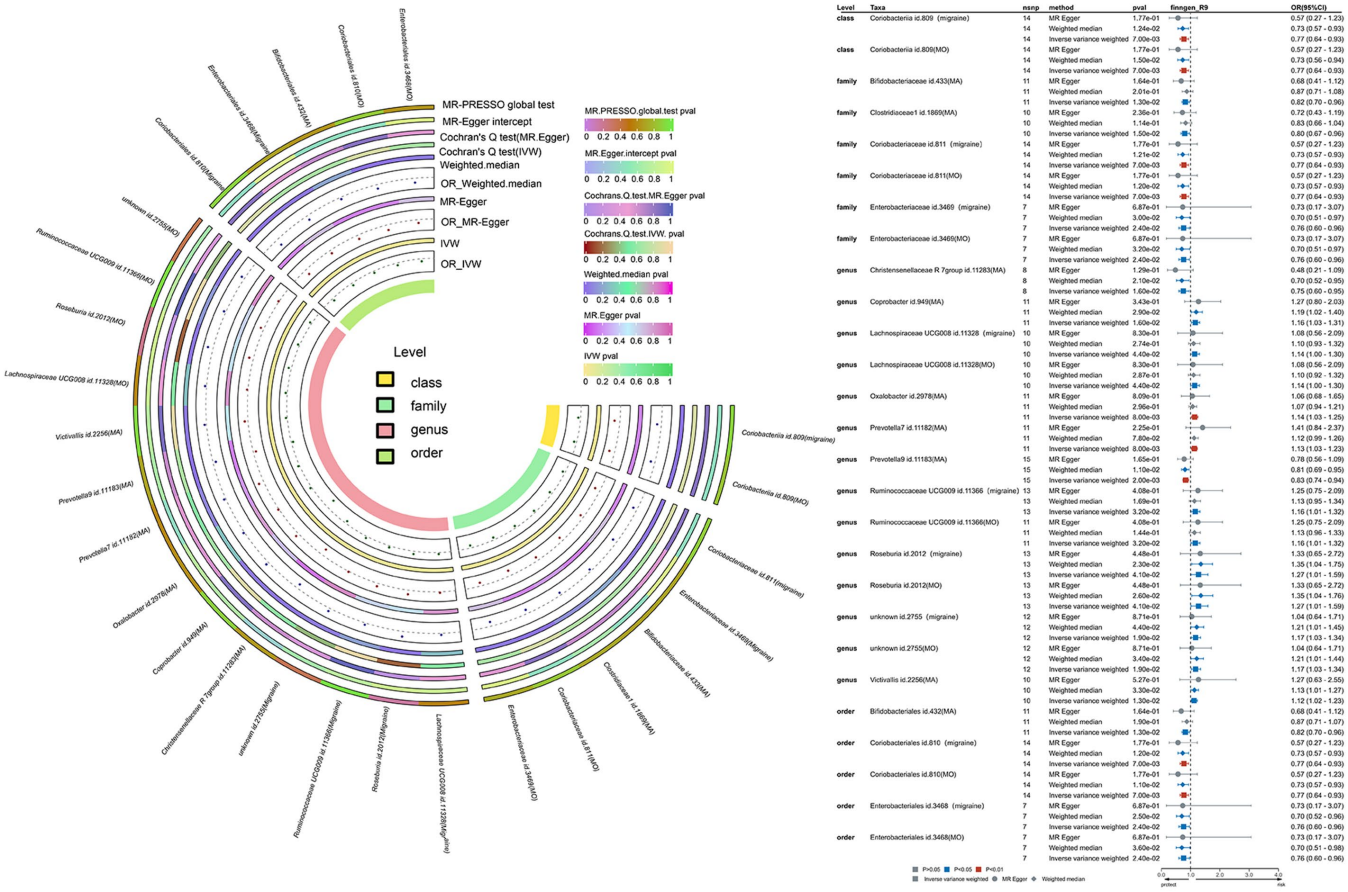
This is a causal analysis of the gut microbiota and migraine disease obtained through a study using the FinnGen study (significant at all loci, *p* < 1 × 10^−5^). MR, Mendelian randomization; IVW, inverse-variance-weighted; MR-PRESSO, MR pleiotropy residual sum and outlier.

**Figure 4:**
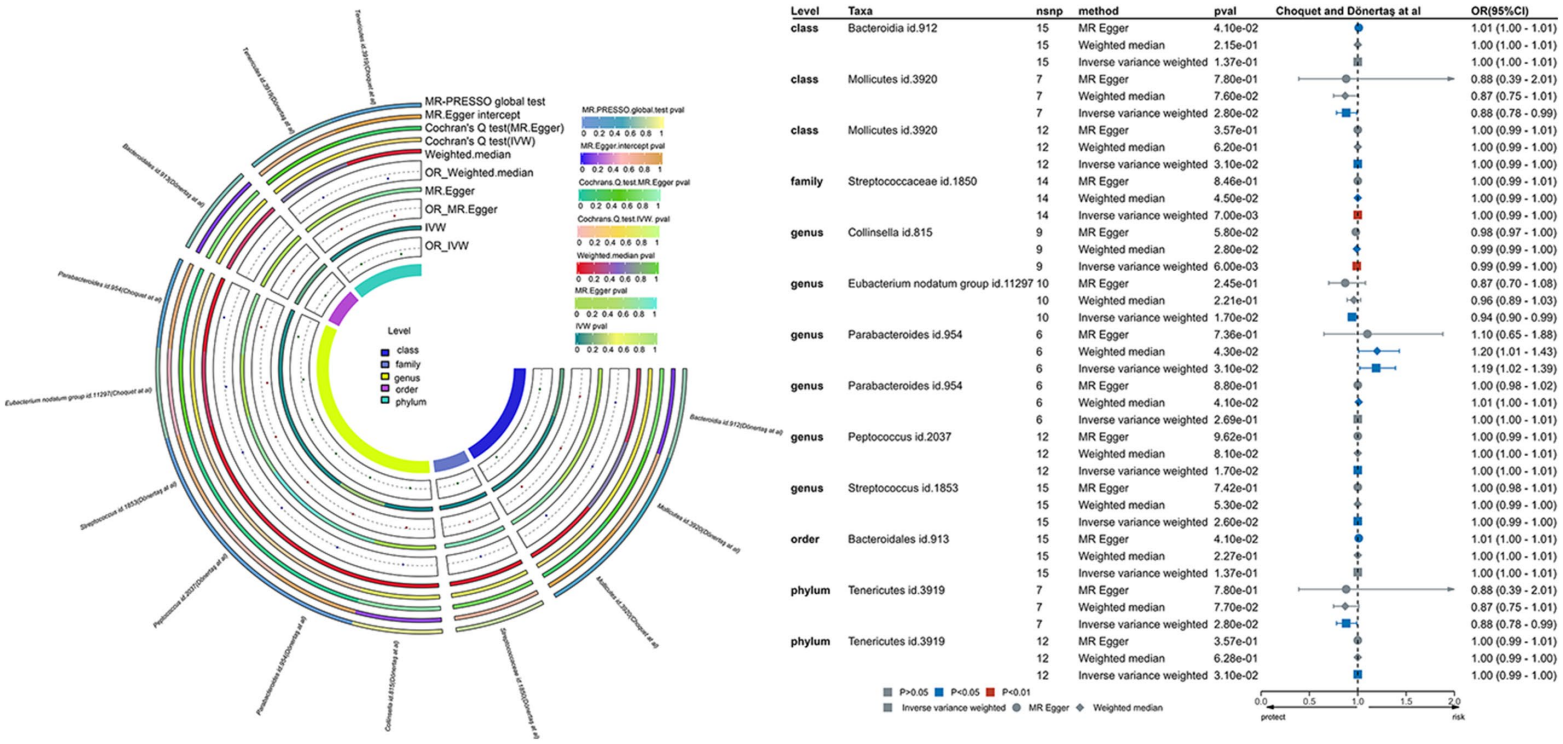
This involves a causal analysis examining the correlation between gut microbiota and migraine disease, as indicated by investigations by Choquet et al. and Dönertaş et al. (statistically significant across all loci, *p* < 1 × 10^–5^). The abbreviations encompass MR for Mendelian randomization, IVW for inverse-variance-weighted, and MR-PRESSO for MR pleiotropy.

To unravel the causal dynamics between each bacterial taxon and migraines, we employed five TSMR methodologies (Supplementary Tables S6–S10). Furthermore, we established potential causal relationships between gut microbiota and migraines using three TSMR approaches (Figures 3, 4). Cross-validation unfolded through the Inverse Variance Weighted (IVW) and Weighted Median (WM) techniques, with a preference for the IVW method as the principal tool for result analysis. A comprehensive examination of the outcomes involved Bonferroni correction to enhance dependability.

Cochran’s Q test was applied to assess heterogeneity among SNPs, focusing on results with a *p*-value exceeding 0.05. Additionally, the outcomes included were required to meet the criteria of MR-PRESSO global test *p* > 0.05 and MR Egger regression *p* > 0.05 to mitigate the risk of horizontal pleiotropy (Supplementary Tables S11, S12). Emphasis was placed on the imperative nature of consistency in the direction of Beta values across all methodologies, as detailed in Supplementary Tables S11, S12. To establish the reliability of our findings, a sensitivity analysis utilizing the leave-one-out approach was executed on identified key bacterial taxa.

#### 3.1.2 FinnGen R9

##### 3.1.2.1 FinnGen_R9_G6_MIGRAINE

The IVW methodology was used to establish causative links between migraines and nine bacterial taxonomic groups. By consolidating these discoveries with cross-validation, the constancy of outcomes for seven bacterial taxonomic groups was confirmed (Supplementary Table S13). Specifically, TSMR analysis revealed a protective impact against migraines for Class Coriobacteriia (OR: 0.77, 95% CI: 0.64–0.93, *p* = 0.007), Family Coriobacteriaceae (OR: 0.77, 95% CI: 0.64–0.93, *p* = 0.007), Family Enterobacteriaceae (OR: 0.76, 95% CI: 0.60–0.96, *p* = 0.024), Order Coriobacteriales (OR: 0.77, 95% CI: 0.64–0.93, *p* = 0.007), and Order Enterobacteriales (OR: 0.76, 95% CI: 0.60–0.96, *p* = 0.024). Conversely, Genus Roseburia (OR: 1.27, 95% CI: 1.01–1.59, *p* = 0.041) and Unknown Genus (OR: 1.17, 95% CI: 1.03–1.34, *p* = 0.019) were identified as significantly amplifying the risk of migraines. After Bonferroni correction, these revelations maintained their causal effects. In the sensitivity analysis, Cochran’s Q test revealed no indications of heterogeneity for the seven identified bacterial taxa. Both the MR-Egger intercept test and MR-PRESSO global test were oblivious to horizontal pleiotropy for these bacterial taxa (Supplementary Table S13). The robustness of the outcomes was further validated through a sensitivity analysis using the leave-one-out approach (Supplementary Figures S1A–F).

##### 3.1.2.2 R9_G6_MIGRAINE_WITH_AURA

Using the IVW methodology, we identified causal associations between migraine with aura and nine bacterial taxonomic groups. Integrating these results with cross-validation, we confirmed the stability of outcomes for four bacterial taxonomic groups (Supplementary Table S14). Specifically, Genus Christensenellaceae R-7 group (OR: 0.75, 95% CI: 0.60–0.95, *p* = 0.016) and Genus Prevotella 9 (OR: 0.83, 95% CI: 0.74–0.94, *p* = 0.002) exhibited a protective effect against migraine with aura, while Genus Coprobacter (OR: 1.16, 95% CI: 1.03–1.31, *p* = 0.016) and Genus Victivallis (OR: 1.12, 95% CI: 1.02–1.23, *p* = 0.013) had an adverse impact. The *p*-values for these findings adhered to Bonferroni’s correction. In the sensitivity analysis, Cochran’s Q test revealed no signs of heterogeneity for the four identified bacterial taxa. Both the MR-Egger intercept test and MR-PRESSO global test were oblivious to horizontal pleiotropy for these bacterial taxa (Supplementary Table S14). The leave-one-out sensitivity analysis plots underscored the robustness of the conclusions (Supplementary Figures S2A–D).

##### 3.1.2.3 R9_G6_MIGRAINE_NO_AURA

The IVW methodology revealed a causal association between migraine without aura and nine bacterial taxonomic groups. Integrating these findings with cross-validation, we confirmed the stability of outcomes for seven bacterial taxonomic groups (Supplementary Table S15). TSMR analysis revealed a protective impact against migraine without aura for Class Coriobacteriia (OR: 0.77, 95% CI: 0.64–0.93, *p* = 0.007), Family Coriobacteriaceae (OR: 0.77, 95% CI: 0.64–0.93, *p* = 0.007), Family Enterobacteriaceae (OR: 0.76, 95% CI: 0.60–0.96, *p* = 0.024), Order Coriobacteriales (OR: 0.77, 95% CI: 0.64–0.93, *p* = 0.007), and Order Enterobacteriales (OR: 0.76, 95% CI: 0.60–0.96, *p* = 0.024). Conversely, Genus Roseburia (OR: 1.27, 95% CI: 1.01–1.59, *p* = 0.041) and Unknown Genus (OR: 1.17, 95% CI: 1.03–1.34, *p* = 0.019) emerged as significantly increasing the risk of migraine without aura. These outcomes align with those derived from the initial migraine Finnish database, further supporting the causal linkage of these seven bacterial taxa with migraine. In the sensitivity analysis, no indications of heterogeneity or horizontal pleiotropy for the seven identified bacterial taxa were detected (Supplementary Table S15). The leave-one-out sensitivity analysis underscored the robustness of the conclusions (Supplementary Figures S3A–G).

### 3.2 UK biobank data

Applying the IVW methodology, we established causal associations between migraines and six bacterial taxonomic groups. Through the integration of these findings with cross-validation, we confirmed the stability of outcomes for two bacterial taxonomic groups (Supplementary Table S16). Specifically, Family Streptococcaceae (OR: 1.00, 95% CI: 0.99–1.00, *p* = 0.007) exhibited a neutral causal connection with migraines, while Genus Collinsella (OR: 0.99, 95% CI: 0.99–1.00, *p* = 0.006) demonstrated a protective impact against migraines.

The *p*-values for the mentioned bacterial taxa fall within the corrected p-value range. In the sensitivity analysis, Cochran’s Q test showed no indications of heterogeneity for Family Streptococcaceae (*p* = 0.756) and Genus Collinsella (*p* = 0.779). The MR-Egger intercept test failed to identify horizontal pleiotropy for Family Streptococcaceae (*p* = 0.637) and Genus Collinsella (*p* = 0.159). Similarly, the MR-PRESSO global test did not detect horizontal pleiotropy for Family Streptococcaceae (*p* = 0.81) and Genus Collinsella (*p* = 0.91; Supplementary Table S16). The durability of the outcomes was underscored through leave-one-out sensitivity analysis (Supplementary Figures S4A,B).

### 3.3 GERA and UK biobank data

Utilizing the IVW methodology, we identified causal associations between migraines and four bacterial taxonomic groups. Integrating these findings with cross-validation, we confirmed that only the results for one bacterial taxonomic group exhibited uniformity (Supplementary Table S17). Our TSMR analysis reveals a clear and statistically significant causal association between Genus Parabacteroides (OR: 1.19, 95% CI: 1.02–1.39, *p* = 0.031) and migraines. Importantly, this conclusion remains robust even after applying Bonferroni correction. In the sensitivity analysis, the absence of indications of heterogeneity for Genus Parabacteroides (*p* = 0.191) in Cochran’s Q test and the failure of the MR-Egger intercept test to identify horizontal pleiotropy (*p* = 0.792). Similarly, the MR-PRESSO global test did not reveal horizontal pleiotropy (*p* = 0.26) for Genus Parabacteroides (Supplementary Table S17). The robustness of the findings is further supported by a leave-one-out sensitivity analysis (Supplementary Figure S5A).

### 3.4 Reverse TSMR analysis

The outcomes of the reverse Mendelian randomization are outlined in Supplementary Tables S18–S31. Upon review of cross-validation findings and sensitivity analysis, it became apparent that the stability of outcomes is retained for a single bacterial taxonomic group (refer to Supplementary Tables S27, S31). Specifics of the leave-one-out sensitivity analysis are illustrated in Supplementary Figure S5B.

### 3.5 Meta-analysis of gut microbiota and migraine

**Figure 5:**
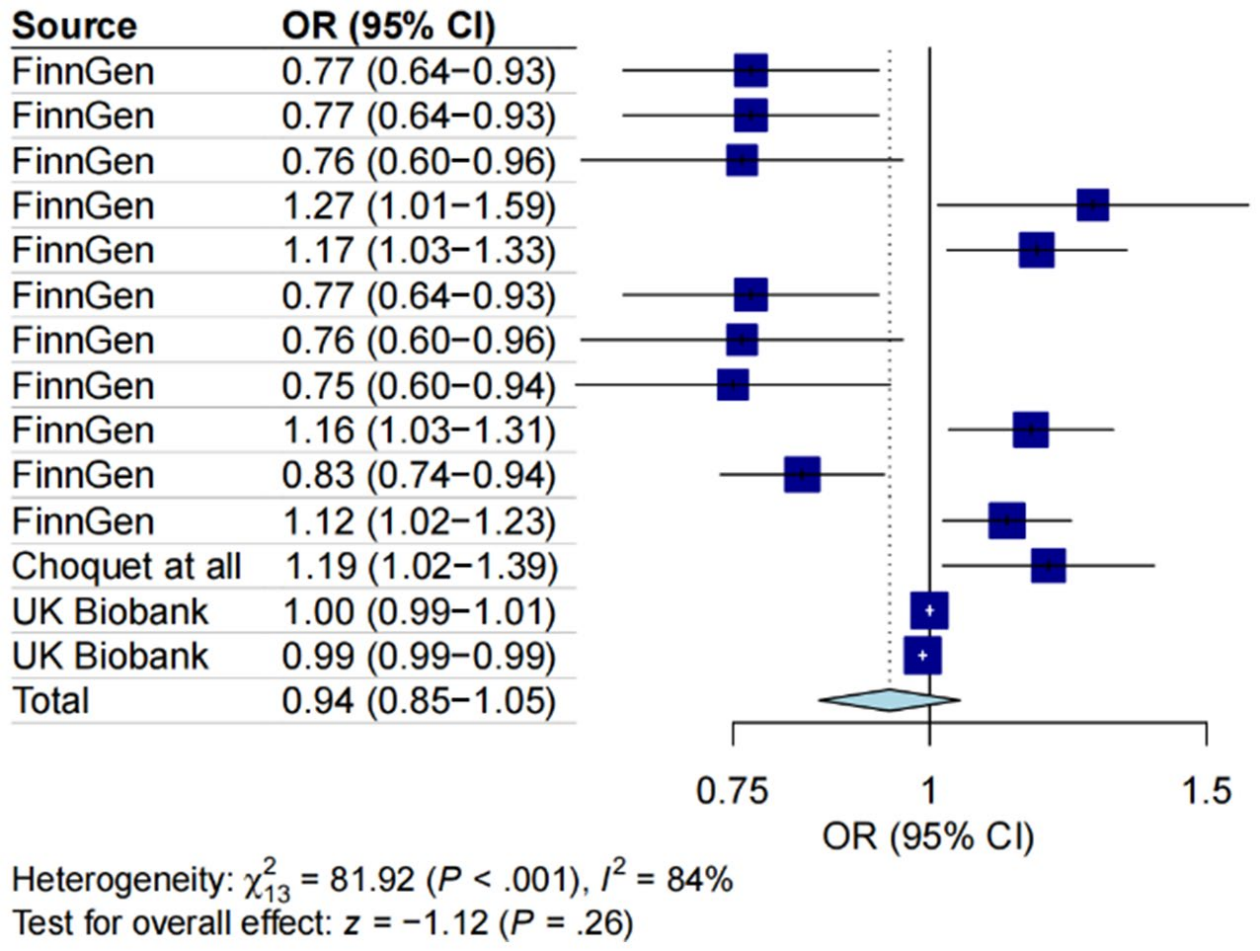
A forest plot of the meta-analysis including five different migraine datasets. The plot illustrates the average genetic predictive effect of Gut microbiota on migraine. The presented odds ratio (OR) and confidence interval (CI) correspond to the average impact of Gut microbiota on migraine. The I2 statistic and chi-square-based Q test were used to assess heterogeneity among studies.

Employing the IVW methodology and integrating cross-validation outcomes, while excluding redundant entries from the Finnish database, a total of 14 observations from five datasets were included in our meta-analysis. The assess heterogeneity among the four datasets, we conducted the I^2^ test, revealing significant heterogeneity (I^2^ = 84%, *p* < 0.001). Consequently, a random-effects model was applied for the meta-analysis. Our results illuminated an adverse impact of gut microbiota on the likelihood of gut microbiota occurrence (OR = 0.97, 95% CI = 0.87–1.08, *p* < 0.001), underscoring the influence of MD on GERD (Figure 5).

## 4 Discussion

**Figure 6:**
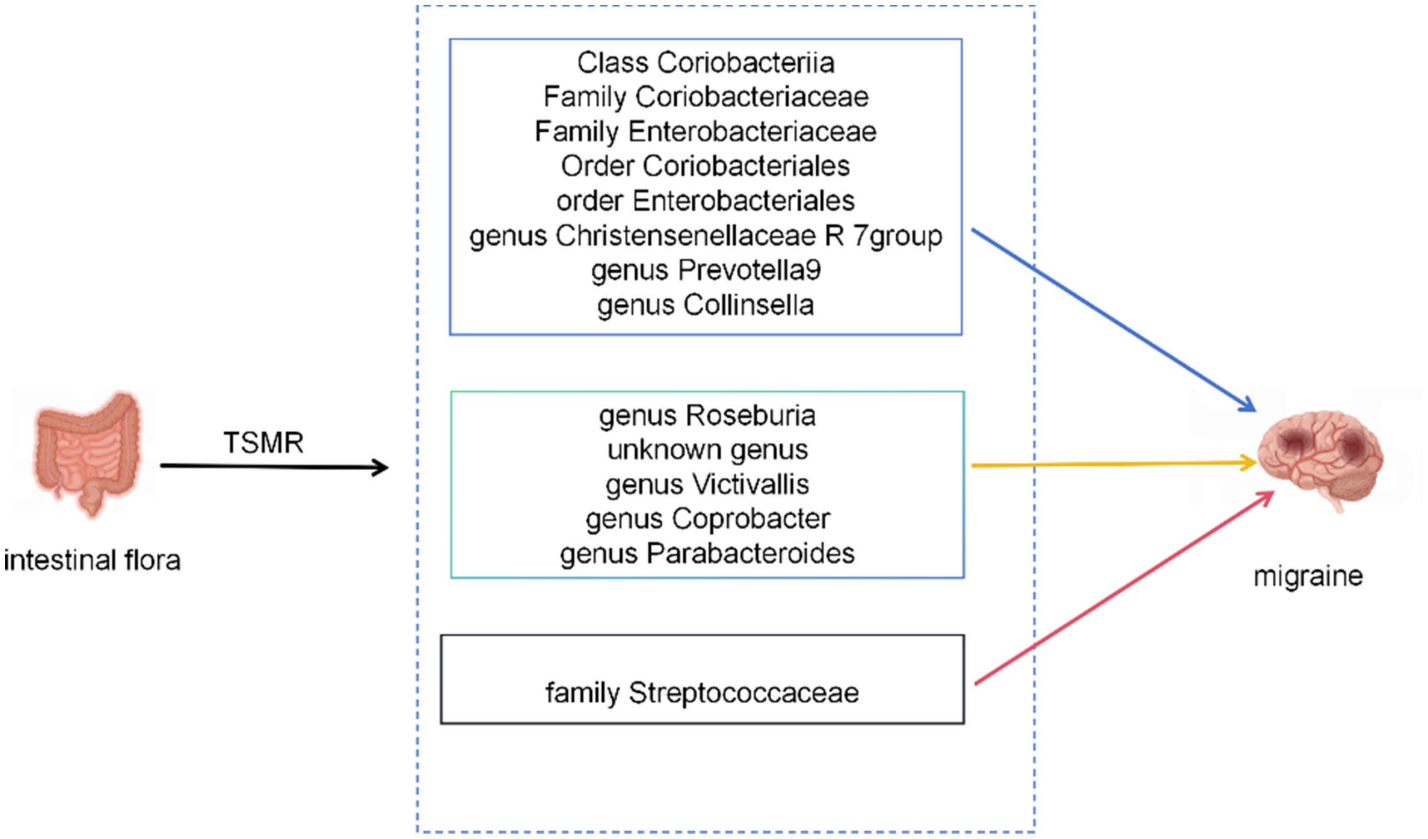
The current MR analysis identified gut microbiota associated with migraines. Blue arrows represent bacterial taxa that are protective factors for the outcome, yellow arrows represent bacterial taxa that are risk factors for the outcome. Red arrows indicate taxa that might be both protective and risk factors.

During TSMR analysis, 14 bacterial features associated with migraines were identified by leveraging extensive aggregated GWAS data (Figure 6).

The current understanding of the mechanisms underlying the interaction between the gastrointestinal tract and the brain in individuals experiencing migraines is currently insufficient. Multiple investigations suggest that this association is influenced by a variety of factors, including inflammatory mediators, gut microbiota, neuropeptides, and other components (43). Of particular significance is the function fulfilled by gut microbiota, impacting the system through the generation of neurotransmitters, inflammatory molecules, and hormones. Moreover, it can directly interact with the nerve endings of the vagus nerve (43). Similarly, the central nervous system can regulate gut microbiota by releasing neuroendocrine peptides, signifying a bidirectional mechanism (44). Substances such as calcitonin gene-related peptide (CGRP), substance P (SP), vasoactive intestinal peptide (VIP), and other compounds are believed to contribute to the bidirectional association between the gastrointestinal tract and the brain. These substances are theorized to exhibit antimicrobial effects on diverse bacterial strains within the gastrointestinal tract, including *Escherichia coli*, *Enterococcus faecalis*, and *Lactobacillus acidophilus* (44). CGRP, playing a pivotal role in the pathophysiology of migraines, is abundantly present in neurons of the trigeminal ganglion, released from peripheral and central nerve endings, and secreted within the trigeminal ganglion. Treatments targeting the functional aspects of CGRP in the peripheral trigeminal system have demonstrated efficacy in managing migraines (45, 46). Consequently, it can be inferred that gut microbiota may confer a significant effect against migraines, aligning with the findings of our investigation.

Numerous studies indicate an elevation in pro-inflammatory cytokines, including IL-1β, IL-6, IL-8, and TN-αduring the interictal phase of migraines. Consequently, the relationship between pro-inflammatory factors and migraines is currently under scrutiny (47, 48). Hirayama et al. discovered that the Collinsella genus can stimulate the production of ursodeoxycholic acid and other secondary bile acids. Ursodeoxycholic acid, in turn, can inhibit pro-inflammatory cytokines such as TNF-α, IL-1β, IL-2, IL-4, and IL-6, at both mRNA and protein levels (49). Moreover, ursodeoxycholic acid exhibits antioxidant and anti-apoptotic properties (49). The order Coriobacteriales, encompassing the genus Collinsella, plays a crucial role in this context (50). From these findings, we deduce that both the genus Collinsella and the order Coriobacteriales may exert a protective effect against migraines. This inference aligns seamlessly with the results of our investigation. Liu et al.’s study, which revealed a comparatively diminished abundance of the Collinsella genus in the gut of migraine patients, provides additional support for this conclusion (51).

At present, there is no direct and well-established evidence connecting the class Coriobacteriia with migraines. Nonetheless, the hierarchical relationship among Class Coriobacteriia, Order Coriobacteriales, and Family Coriobacteriaceae is well recognized. The classification begins broadly with Class Coriobacteriia and becomes more specific through Order Coriobacteriales to Family Coriobacteriaceae, each being nested within the previous, revealing a tight genetic and functional connection (52). Therefore, we propose that Class Coriobacteriia and Family Coriobacteriaceae could have a relationship with migraines. Previous studies suggest that Coriobacteriaceae contributes to host bile acid and lipid metabolism (53), further reinforcing our conclusions.

Recent studies have revealed a significant connection between migraines and irritable bowel syndrome (IBS) (54). Among individuals with IBS, approximately 25%–50% report encountering migraines or headaches, a notable contrast to the 4%–19% prevalence in the control group (55). The odds ratio for the simultaneous occurrence of IBS and migraines or headaches among IBS individuals stands at 2.66 (10), indicating an elevated likelihood of migraines in this subgroup. Several investigations have highlighted that, within the healthy control cohort, the proportional prevalence of Christensenellaceae surpasses that observed in individuals with IBS. This distinction may be linked to inflammatory processes and an expedited transit time in IBS patients (56). Additionally, a heightened proportional prevalence of Christensenellaceae has been documented in patients diagnosed with Parkinson’s disease and multiple sclerosis relative to their healthy counterparts (57–60). This nuanced observation supports the proposition that Christensenellaceae, by shaping the gut milieu in individuals with IBS, potentially contributes to the manifestation of migraines.

In alignment with the aforementioned investigations, the research of Ornello et al. (61) implies an increased susceptibility to migraines and chronic migraines in individuals grappling with obesity. Goodrich et al. (62) observed a significant enrichment of Christensenellaceae in individuals maintaining a normal BMI (18.5–24.9) compared to those classified as obese (BMI ≥ 30). Furthermore, the prevalence of Christensenellaceae exhibited a surge following weight loss induced by dietary modifications (63). This suggests a plausible association between Christensenellaceae and migraines, presenting a novel perspective on migraine management for individuals contending with obesity. Our study similarly posits a causal connection between Christensenellaceae and migraines, underscoring the imperative for supplementary experimental affirmation.

Research into obesity-related conditions indicates an increase in the abundance of Enterobacteriaceae within the gut microbiota of obese subjects. When weight loss occurs, the Enterobacteriaceae population decreases. In contrast, transferring Enterobacteriaceae from obese humans into mice led to increased fat formation in the animals (64). This finding implies a link between Enterobacteriaceae and obesity. Given the earlier discussion on the correlation between obesity and migraines, it can be hypothesized that Enterobacteriaceae could also be associated with migraines.

He et al.’s investigation uncovers a causal link between the genera Prevotella9 (OR = 0.82, 95%CI = 0.72–0.94, *p* = 0.004), Roseburia (OR = 1.15, 95%CI = 1.02–1.30, *p* = 0.0234), and Coprobacter (OR = 1.09, 95%CI = 1.01–1.17, *p* = 0.0347) and migraines. Prevotella9 is suggested to confer a protective effect against migraines, while Roseburia and Coprobacter are implicated in an increased risk of migraines (21). These outcomes resonate with our study findings. Nonetheless, the precise mechanisms remain elusive, necessitating additional investigation for validation.

Parabacteroides, a collection of anaerobic Gram-negative bacteria commonly found in the human gastrointestinal tract, possess the ability to induce pro-inflammatory responses through lipopolysaccharide (LPS) and the metabolic byproduct succinic acid (65). Research has illuminated an increased prevalence of Parabacteroides in individuals experiencing migraines in conjunction with irritable bowel syndrome (IBS) (51). Therefore, we propose that Parabacteroides may exert an unfavorable influence on migraines, aligning with our investigation. While current research has not specifically examined the associations between the family Streptococcaceae, unidentified genera, and the genus Victivallis with migraines, this gap presents a valuable opportunity for future studies.

Our experiments have yielded several notable advantages. Firstly, this investigation reaffirms the causal link between gut microbiota and migraines through bidirectional TSMR analysis. This analysis remains robust against confounding factors or reverse causation. Secondly, we set stringent criteria for selecting instrumental variables, differentiating our approach from similar studies. We only consider causation plausible when two or more TSMR methods support the relationship. Thirdly, we utilized data from five GWAS datasets, distinct from those databases previously used in studies on gut microbiota and migraines. This not only strengthens the credibility and stability of our MR research but also marks a significant enhancement in evaluating the causal connection between gut microbiota and migraines. Moreover, we conducted the first-ever meta-analysis of results for this type of study, further reinforcing the robustness of our research outcomes. Fourthly, this work establishes the foundation for scrutinizing the gut-brain axis from a genetic standpoint. Through TSMR analysis, we identified 14 bacterial taxa linked to migraines. These crucial bacterial taxa could pave the way for future research on migraine treatment by promoting beneficial bacteria and inhibiting the growth of harmful bacteria to prevent and treat migraines.

This study has specific limitations. Firstly, the gut microbiota GWAS statistics include a limited number of instrumental variables, and there is an insufficient count of associated loci for bacterial taxa. To address this, we selected a more lenient association threshold to ensure an adequate number of SNPs. Secondly, confirming overlapping participants in the GWAS data related to exposures and outcomes central to this study is impossible. Thirdly, the original studies mainly focused on European blood populations and lacked demographic data, hindering subgroup analyses based on factors such as age. Moreover, our findings require additional validation through clinical and foundational research. In future studies, we aim to increase the sample size, conduct collaborative experiments, and more precisely explore the relationship between gut microbiota and migraines at the species level.

Recent studies involving European populations have shown that modifications to dietary habits can influence the gut microbiome, impacting the production of short-chain fatty acids (SCFAs) (66). The research indicates that, compared to the Canadian Diet (CanDiet), the Mediterranean Diet (MedDiet) might produce higher levels of SCFAs, which are known for their neuroprotective effects (43). This leads to the hypothesis that dietary changes could help reduce migraine episodes. Additional research points to the potential benefits of a high omega-3/low omega-6 diet, a low glycemic index diet, and supplementation of probiotics and vitamin D, as these interventions may improve migraines by promoting SCFAs production and managing inflammation (43).

## 5 Conclusion

In summary, our use of MR analysis provides new evidence supporting the causal link between gut microbiota and migraines. Our study suggests that specific taxonomic groups, such as Class Coriobacteriia, Family Coriobacteriaceae, Family Enterobacteriaceae, Order Coriobacteriales, and others, might exert a notable influence on migraines. The implications of our findings extend to the potential development of innovative interventions and treatments for migraines. Nevertheless, additional validation is required through clinical trials or foundational experiments.

## Data availability statement

The original contributions presented in the study are included in the article/Supplementary material, further inquiries can be directed to the corresponding authors.

## Author contributions

XZ: Writing – review & editing, Writing – original draft, Visualization, Formal analysis, Data curation. YD: Writing – review & editing, Data curation. MJ: Writing – review & editing, Data curation. SZ: Writing – review & editing, Data curation. LW: Writing – review & editing, Formal analysis, Data curation. XH: Writing – review & editing, Visualization, Formal analysis, Data curation.

## Footnotes

^1^https://mibiogen.gcc.rug.nl/

^2^https://www.finngen.fi/en/access_results
