# Supplementary material for: A thorough investigation into the correlation between migraines and the gut microbiome: an in-depth analysis using Mendelian randomization studies
Source: Front Neurol. 2024 Jul 2;15:1356974. doi: 10.3389/fneur.2024.1356974 (PMC11250663; doi:10.3389/fneur.2024.1356974)
Supplement: Supplementary file 1 [file Data_Sheet_1.ZIP › Supplementary_Material/Supplementary_Material.docx]

Supplementary Material

A thorough investigation into the correlation between migraines and the gut microbiome: An in-depth analysis using Mendelian Randomization Studies

Xuege Zang*, Yongkun Du, Mengshu Jiang

*** Correspondence:** Libo Wang, [wang_libo@jlu.edu.cn](mailto:wang_libo@jlu.edu.cn); Xuemei Han, [hxm@jlu.edu.cn](mailto:hxm@jlu.edu.cn)

# Supplementary Data

**Description**

**Table S1** Characteristics of the genetic IVs used in TSMR analysis (finngen_R9_G6_MIGRAINE, P<1.00E-05).

**Table S2** Characteristics of the genetic IVs used in TSMR analysis (R9_G6_MIGRAINE_WITH_AURA, P<1.00E-05).

**Table S3** Characteristics of the genetic IVs used in TSMR analysis(R9_G6_MIGRAINE_NO_AURA, P<1.00E-05).

**Table S4** Characteristics of the genetic IVs used in TSMR analysis(UK Biobank data, P<1.00E-05).

**Table S5** Characteristics of the genetic IVs used in TSMR analysis(GERA and UK Biobank data, P<1.00E-05).

**Table S6** Casual effects of TSMR Analysis between gut microbiota and migraine (finngen_R9_G6_MIGRAINE, P<1.00E-05).

**Table S7** Casual effects of TSMR Analysis between gut microbiota and migraine (R9_G6_MIGRAINE_WITH_AURA, P<1.00E-05).

**Table S8** Casual effects of TSMR Analysis between gut microbiota and migraine (R9_G6_MIGRAINE_NO_AURA,P<1.00E-05).

**Table S9** Casual effects of TSMR Analysis between gut microbiota and migraine (UK Biobank data, P<1.00E-05).

**Table S10** Casual effects of TSMR Analysis between gut microbiota and migraine (GERA and UK Biobank data, P<1.00E-05).

**Table S11** Positive results of Mendelian randomization analysis for three approaches(FINNGEN-R9).

**Table S12** Positive results of Mendelian randomization analysis for three approaches(Choquet at al and Dönertaş at al).

**Table S13** Gut Microbiota and Migraine: Mendelian Randomization Cross-Validation Results, Heterogeneity, and Sensitivity Analysis Results(finngen_R9_G6_MIGRAINE).

**Table S14** Gut Microbiota and Migraine: Mendelian Randomization Cross-Validation Results, Heterogeneity, and Sensitivity Analysis Results(R9_G6_MIGRAINE_WITH_AURA).

**Table S15** Gut Microbiota and Migraine: Mendelian Randomization Cross-Validation Results, Heterogeneity, and Sensitivity Analysis Results(R9_G6_MIGRAINE_NO_AURA).

**Table S16** Gut Microbiota and Migraine: Mendelian Randomization Cross-Validation Results, Heterogeneity, and Sensitivity Analysis Results(UK Biobank).

**Table S17** Mendelian Randomization Cross-Validation Results, Heterogeneity, and Sensitivity Analysis Results(GERA and UK Biobank data).

**Table S18** Characteristics of the genetic IVs used in TSMR analysis (P<5E-08, finngen_R9_G6_MIGRAINE ).

**Table S19** Characteristics of the genetic IVs used in TSMR analysis(P<5E-08, R9_G6_MIGRAINE_WITH_AURA).

**Table S20** Characteristics of the genetic IVs used in TSMR analysis(P<5E-08, R9_G6_MIGRAINE_NO_AURA).

**Table S21** Characteristics of the genetic IVs used in TSMR analysis(P<5E-08, UK Biobank data).

**Table S22** Characteristics of the genetic IVs used in TSMR analysis(P<5E-08, GERA and UK Biobank data).

**Table S23** Casual effects of TSMR Analysis between migraine and gut microbiota (P<5.00E-08,finngen_R9_G6_MIGRAINE ).

**Table S24** Casual effects of TSMR Analysis between migraine and gut microbiota(P<5.00E-08, R9_G6_MIGRAINE_WITH_AURA).

**Table S25** Casual effects of TSMR Analysis between migraine and gut microbiota(P<5.00E-08, R9_G6_MIGRAINE_NO_AURA).

**Table S26** Casual effects of TSMR Analysis between migraine and gut microbiota(P<5.00E-08, UK Biobank data).

**Table S27** Casual effects of TSMR Analysis between migraine and gut microbiota(P<5.00E-08, GERA and UK Biobank data).

**Table S28** Sensitivity analysis of positive Mendelian randomization results(P<5.00E-08, finngen_R9_G6_MIGRAINE).

**Table S29** Sensitivity analysis of positive Mendelian randomization results(P<5.00E-08, R9_G6_MIGRAINE_NO_AURA).

**Table S30** Sensitivity analysis of positive Mendelian randomization results(P<5.00E-08, UK Biobank data).

**Table S31** Sensitivity analysis of positive Mendelian randomization results(P<5.00E-08, GERA and UK Biobank data).

**Figure S1(A-G)** Gut Microbiota and Migraine: Mendelian Randomization Analysis of Positive Results, Leave-One-Out Sensitivity Analysis(finngen_R9_G6_MIGRAINE).

**Figure S2(A-D)** Gut Microbiota and Migraine: Mendelian Randomization Analysis of Positive Results, Leave-One-Out Sensitivity Analysis(R9_G6_MIGRAINE_WITH_AURA).

**Figure S3(A-G)** Gut Microbiota and Migraine: Mendelian Randomization Analysis of Positive Results, Leave-One-Out Sensitivity Analysis(R9_G6_MIGRAINE_NO_AURA).

**Figure S4(A-B)** Gut Microbiota and Migraine: Mendelian Randomization Analysis of Positive Results, Leave-One-Out Sensitivity Analysis(UK Biobank data).

**Figure S5(A)** Gut Microbiota and Migraine: Mendelian Randomization Analysis of Positive Results, Leave-One-Out Sensitivity Analysis(GERA and UK Biobank data). **Figure S5(B)** Reverse Mendelian Randomization Positive Results Leave-One-Out Sensitivity Analysis.

# 2 Supplementary Figures and Tables

## 2.1 Supplementary Figures

For details, see Figures S1-S5.

**
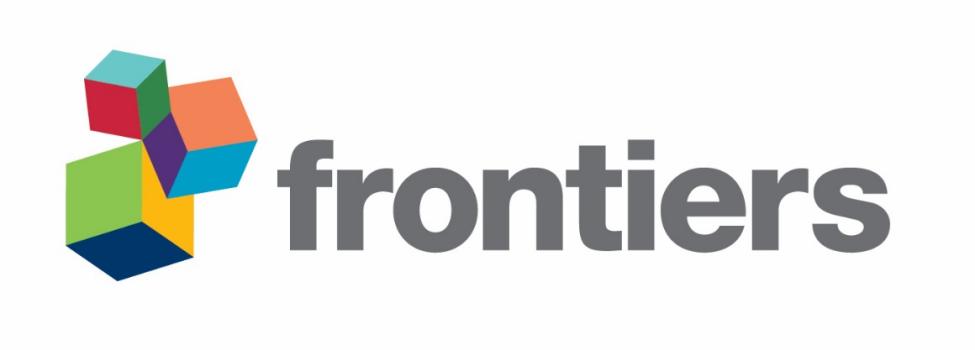
**

**2.2 Supplementary Tables**

For details, see Tables S1-S31.
