# Supplementary material for: A thorough investigation into the correlation between migraines and the gut microbiome: an in-depth analysis using Mendelian randomization studies
Source: Front Neurol. 2024 Jul 2;15:1356974. doi: 10.3389/fneur.2024.1356974 (PMC11250663; doi:10.3389/fneur.2024.1356974)
Supplement: Supplementary file 1 [file Data_Sheet_1.ZIP › Supplementary_Material/Figures S1-S5.doc]

FIG1(A-G): Gut Microbiota and Migraine: Mendelian Randomization Analysis of Positive Results, Leave-One-Out Sensitivity Analysis(finngen_R9_G6_MIGRAINE).

FIG2(A-D): Gut Microbiota and Migraine: Mendelian Randomization Analysis of Positive Results, Leave-One-Out Sensitivity Analysis(R9_G6_MIGRAINE_WITH_AURA).

FIG3(A-G): Gut Microbiota and Migraine: Mendelian Randomization Analysis of Positive Results, Leave-One-Out Sensitivity Analysis(R9_G6_MIGRAINE_NO_AURA).

FIG4(A-B): Gut Microbiota and Migraine: Mendelian Randomization Analysis of Positive Results, Leave-One-Out Sensitivity Analysis(UK Biobank data).

FIG5(A): Gut Microbiota and Migraine: Mendelian Randomization Analysis of Positive Results, Leave-One-Out Sensitivity Analysis(GERA and UK Biobank data). FIG5(B): Reverse Mendelian Randomization Positive Results Leave-One-Out Sensitivity Analysis.
